# Genomic characteristics and antibiotic resistance profiles of monophasic *Salmonella* Typhimurium in Shaanxi Province, China

**DOI:** 10.3389/fmicb.2025.1565631

**Published:** 2025-04-11

**Authors:** Yi Shi, Yuguo Liu, Shen Li, Songwen Wu, Guozhu Ma, Yang Luan, Junjun Zhang, Yali Chen, Wanjing Liu, Tuo Shen, Caiqiao Wang, Jiru Xu

**Affiliations:** ^1^Department of Microbiology and Immunology, School of Medicine, Xi'an Jiaotong University, Xi'an, China; ^2^Shaanxi Center for Disease Control and Prevention, Xi’an, China; ^3^Department of Rehabilitation Medicine, Zhujiang Hospital, Southern Medical University, Guangzhou, China; ^4^Department of Public Health, Xi’an Medical University, Xi’an, China; ^5^Xi’an Center for Disease Control and Prevention, Xi’an, China; ^6^Yanta Center for Disease Control and Prevention, Xi’an, China; ^7^Hanzhong Center for Disease Control and Prevention, Hanzhong, China; ^8^Ankang Center for Disease Control and Prevention, Ankang, China; ^9^Weinan Center for Disease Control and Prevention, Weinan, China; ^10^Yulin Center for Disease Control and Prevention, Yulin, China

**Keywords:** *salmonella*, antimicrobial resistance, virulence factors, plasmid replicons, WGS, SNP, PFGE, MLST

## Abstract

**Introduction:**

Monophasic *Salmonella* Typhimurium, characterized by the absence of phase II flagellar antigens, has become increasingly prevalent as a foodborne pathogen, raising significant public health concerns due to its multidrug resistance. This study investigated the genomic characteristics and antibiotic resistance profiles of the monophasic *Salmonella* Typhimurium strains isolated from patients and food sources in Shaanxi Province, China.

**Methods:**

A total of 58 strains were collected between 2020 and 2021, with 4 strains isolated from food and 54 from patients. Whole genome sequencing was performed to assess genomic features. Antimicrobial susceptibility was tested against 17 antimicrobial agents using the broth dilution method, while pulsed-field gel electrophoresis (PFGE) and multi-locus sequence typing were employed for genetic characterization and epidemiological analysis. Phylogenetic analysis was conducted using single nucleotide polymorphism clustering.

**Results and discussion:**

Our results revealed that all the strains belonged to the ST34 and did not carry virulence genes on pSLT (NC_003277). There were 12 strains carrying the *STM2757* gene. The isolates exhibited a considerable diversity in PFGE subtypes. Phenotypic antimicrobial resistance showed that the strains were most resistant to tetracycline (94.34%; 50/53) and ampicillin (94.34%; 50/53), followed by streptomycin (88.68%; 47/53) and ampicillin/sulbactam (64.15%; 34/53). Resistance gene prediction highlighted the presence of 64 distinct genes, with *aac(6’)-Iaa* found in all strains (100%) and *tet*(B) in 93.1% of strains. Notably, the *floR* gene, relevant for resistance to phenicols, was observed in 44.83% of isolates. Genomic analysis revealed that 96.55% of strains were positive for the *sodC1* virulence gene, whereas only 10.34% carried the *sopE* gene. The most plasmid replicon was IncQ1 (84.48%; 49/58), followed by IncHI2 (32.76%; 19/58) and IncHI2A (32.76%; 19/58). Single nucleotide polymorphism analysis showed that 2 strains were clustered together with SRR17830210 (UK outbreak isolate) with a bootstrap value of 0.949. There were only 12 allelic differences between SNXiAn21SAL011 and the reference strain. Conclusively, the monophasic *Salmonella* Typhimurium ST34 strains in Shaanxi Province demonstrated unique genomic and antimicrobial resistance traits. This study may help to prevent outbreaks and rationalize salmonellosis antimicrobial therapeutics.

## 1 Introduction

*Salmonella* is among the most prevalent foodborne pathogens. Specifically, nontyphoidal *Salmonella* remains an important etiology of diarrhea, accounting for an estimated 197.35 million episodes (95% uncertainty interval: 127.37 million to 299.20 million) and resulting in approximately 84,799 deaths (95% uncertainty interval: 46,201 to 144,935) annually worldwide (Woh et al., 2021). The *Salmonella* genus includes two species and six subspecies, and there are approximately 2,659 serotypes (Monte and Sellera, 2020). Among these serotypes, *Salmonella enterica* serovar Typhimurium and Enteritidis are most frequently associated with human diseases (Ritter et al., 2019).

The antigenic formula of monophasic *Salmonella* Typhimurium is 4,[5],12:i:-, representing a variant of *Salmonella* Typhimurium. This variant was first isolated from poultry in Portugal during the late 1980s and has since disseminated extensively across Europe, America, Australia, and Asia (Ingle et al., 2021). The primary characteristic of monophasic variants is the absence of phase II flagellar antigens. Potential molecular mechanisms for this absence include the deletion of the *fljB* gene, which encodes the phase II flagellar antigen, or the deletion of related regulatory genes such as *fljA* and *hin*. These deletions result in a serotype change from 4,[5],12:i:1,2 to 4,12:i:- or 4,5,12:i:- (Bugarel et al., 2012). Multiple-locus sequence typing (MLST) analysis has revealed that this serotype predominantly consists of ST19, ST34, and ST313, with ST34 being the most significant type (Van Puyvelde et al., 2019).

In recent years, monophasic strains of *Salmonella enterica* have rapidly spread, resulting in worldwide outbreaks and a concomitant increase in multidrug resistance, which poses significant public health concerns (Larkin et al., 2022; Lund et al., 2022). This serotype currently ranks third among *Salmonella* infections in the European Union and fifth in the United States (National Center for Emerging and Zoonotic Infectious Diseases (U.S.), n.d.; European Food Safety Authority and European Centre for Disease Prevention and Control, 2021). Since December 2021, several European countries have reported suspected clusters of infections with ST34 monophasic *Salmonella enterica* Typhimurium, which have been associated with the consumption of contaminated chocolate products (World Health Organization, 2022). Notably, monophasic *Salmonella* Typhimurium has emerged as the third most prevalent serotype in China (Bai et al., 2022), thereby raising significant public health concerns. It has been reported that monophasic *Salmonella* Typhimurium has become the dominant serotype of human *Salmonella* isolates in Guangdong and Guangxi province in China (Ke et al., 2018; Zeng et al., 2020). However, research specifically focusing on monophasic *Salmonella* Typhimurium in Shaanxi Province remains limited.

This study investigated the genomic characteristics and antibiotic resistance profiles of 58 strains of monophasic *Salmonella* Typhimurium, which were collected from various sources, including patients with diarrhea-related illnesses and food samples. Whole genome sequencing (WGS), bioinformatics analysis, pulsed-field gel electrophoresis (PFGE), single nucleotide polymorphism (SNP) analysis, and antimicrobial susceptibility testing were performed on these strains. Our findings elucidate the molecular epidemiological characteristics of local monophasic *Salmonella* Typhimurium, which may aid in preventing outbreaks and optimizing antimicrobial therapies for salmonellosis.

## 2 Methods

### 2.1 Sample collection

This study collected 58 strains of monophasic *Salmonella* Typhimurium isolated from 6 cities (Xi’an, Xianyang, Weinan, Hanzhong, Ankang, and Yulin) during the years 2020 and 2021. Among them, 4 strains were obtained from food sources (one cooked meat product and three samples of raw pork), while 54 strains were isolated from patients with diarrhea-related illnesses. This study uses strains obtained from Xi’an Center for Disease Control and Prevention (CDC), Yanta CDC, Hanzhong CDC, Ankang CDC, Weinan CDC, and Yulin CDC. These institutions did not require the study to be reviewed or approved by an ethics committee because these strains were gifted from these institutions.

### 2.2 PFGE

According to the China CDC Pathogen Identification Net (CCPIN) protocol, a standardized PFGE typing method was used for the molecular subtyping of *Salmonella*. In brief, *Salmonella* isolates were plated onto Columbia agar supplemented with 5% sheep blood (HuanKai Microbial, Guangzhou, China) and incubated at 37°C for 24 h. The concentration of the *Salmonella* suspension was adjusted to 4.5 McFarland units. The *Salmonella enterica* serotype Braenderup strain (H9812) served as a molecular weight marker (Ribot et al., 2006). Plugs were created by mixing 400 μL of 1% Seakem Gold (Lonza, Rockland, United States) with 400 μL of 1% SDS (Solarbio, Beijing, China) in the bacterial suspension. This mixture was digested with proteinase K at a concentration of 20 mg/mL (Merck, Darmstadt, Germany) at 54°C for 2 h. Digestion was subsequently performed at 37°C for 2 h using the XbaI enzyme (50 U) (Takara, Dalian, China). Additionally, 1.5 g of Seakem Gold agarose was dissolved in 150 mL of 0.5 × TBE (TianGen, Beijing, China). Gel electrophoresis was performed on the CHEF Mapper (Bio-Rad Laboratories, Hercules, United States), with molecular weight parameters set to 30–700 kb and an electrophoresis time of 18 h at 14°C. Other parameters remained at default settings. After electrophoresis, the gel was stained with GelRed (Bosunlife, Beijing, China) for 20 min. The dendrogram was automatically generated online by the CCPIN platform. The software deployment and background parameters were uniformly set by the China CDC, ensuring consistency in the analysis.

### 2.3 Antimicrobial susceptibility testing

The antimicrobial resistance of the isolated *Salmonella* strains was phenotypically evaluated using the broth dilution method. The minimum inhibitory concentration of a panel of 17 antimicrobial agents from 8 antimicrobial classes was determined. The results were interpreted according to the most recent Clinical Laboratory Standard Institute guidelines (2024) (CLSI, 2024). The tested antimicrobial agents included phenicols (chloramphenicol, 4–32 μg/mL), sulphonamide and trimethoprim (trimethoprim-sulfamethoxazole, 0.5–8 μg/mL), polymyxins (colistin, 0.25–8 μg/mL), beta-lactamase inhibitors (ertapenem, 0.25–8 μg/mL; meropenem, 0.125–2 μg/mL; cefotaxime, 0.25–16 μg/mL; ceftazidime, 0.25–16 μg/mL; ceftazidime/avibactam, 0.25/4–8/4 μg/mL; ampicillin, 2–32 μg/mL; ampicillin/sulbactam, 2–32 μg/mL), tetracyclines (tetracycline, 1–16 μg/mL; tigecycline, 0.25–8 μg/mL), fluoroquinolones (ciprofloxacin, 0.015–2 μg/mL; nalidixic acid, 4–32 μg/mL), macrolides (azithromycin, 2–64 μg/mL), and aminoglycosides (amikacin, 4–64 μg/mL; streptomycin, 4–32 μg/mL). *Escherichia coli* ATCC 25922 and *Klebsiella pneumoniae* ATCC700603 served as the quality control strains. Heatmap visualization was performed using the R (version 4.4.2) programming language.

### 2.4 WGS

Genomic DNA was extracted using the QIAamp DNA Mini Kit (QIAGEN, Hilden, Germany). WGS was conducted on the Illumina MiniSeq platform (Illumina, San Diego, CA, United States) using a 150-base pair paired-end strategy. The genomic DNA library was constructed using the Nextera XT DNA Library Preparation Kit (MicroFuture, Beijing, China). Subsequently, genomic sequencing was performed using the MiniSeq High Output Kit (300 cycles) (MicroFuture, Beijing, China).

### 2.5 Bioinformatic analysis

The raw sequence reads were checked for quality and assembled using SPAdes v3.15.2 (Bankevich et al., 2012). The gene sequences of *fljA*, *fljB*, *hin*, *iroB*, and *STM2757* were retrieved from the National Center for Biotechnology Information, and sequence alignment was performed using BLAST 2.12.0+. Virulence gene prediction was conducted based on the virulence factors database (Wu et al., 2005). In silico serotyping of *Salmonella* strains and antimicrobial resistance genes were detected using pipelines on the CCPIN platform. The MLST and the plasmid replicons of the strains were screened and determined by using the MLST 2.0 server and Plasmid Finder 2.1 at the Center for Genomic Epidemiology (Larsen et al., 2012; Carattoli et al., 2014), respectively.

### 2.6 Phylogenetic relationship analysis

The genome sequences of four representative strains of *Salmonella enterica* (UK outbreak isolates), specifically SRR17830210, SRR18021617, SRR18488397, and SRR18590198, were retrieved from the European Bioinformatics Institute/European Nucleotide Archive database. Using SRR17830210 as the reference genome and the CSI Phylogeny 1.4 tool at the Center for Genomic Epidemiology (Kaas et al., 2014), SNP clustering analysis was performed on 30 *Salmonella enterica* strains isolated in 2021. These strains were selected because of their relevance to the European multi-country outbreak of monophasic *Salmonella* Typhimurium ST34 linked to chocolate products that occurred between the end of 2021 and the beginning of 2022, allowing for a more focused analysis of epidemiologically significant isolates. Finally, the resulting phylogenetic tree was visualized using the iTOL software for better interpretation and communication of the evolutionary relationships. The Ridom SeqSphere+ (version 10.0.4) was used for core genome MLST (Junemann et al., 2013).

## 3 Results

### 3.1 Basic genetic characteristics

To determine the genetic characterization, basic gene analysis was performed. As shown in Table 1, the 58 strains of monophasic *Salmonella* Typhimurium did not carry the *fljB* gene or virulence genes on the virulence plasmid of *Salmonella* Typhimurium (pSLT) (NC_003277). One strain carried *fljA*, one strain carried *hin*, 57 strains carried *iroB*, and 12 strains carried the *STM2757* gene (Table 1).

**Table 1:** Genetic characteristics of 58 strains of monophasic *Salmonella* Typhimurium.

	*fljA*	*fljB*	*hin*	*iroB*	*STM2757*	Genes on pSLT
Absent	57	58	57	1	46	58
Present	1	0	1	57	12	0

### 3.2 PFGE and MLST sequence types

**Figure 1:**
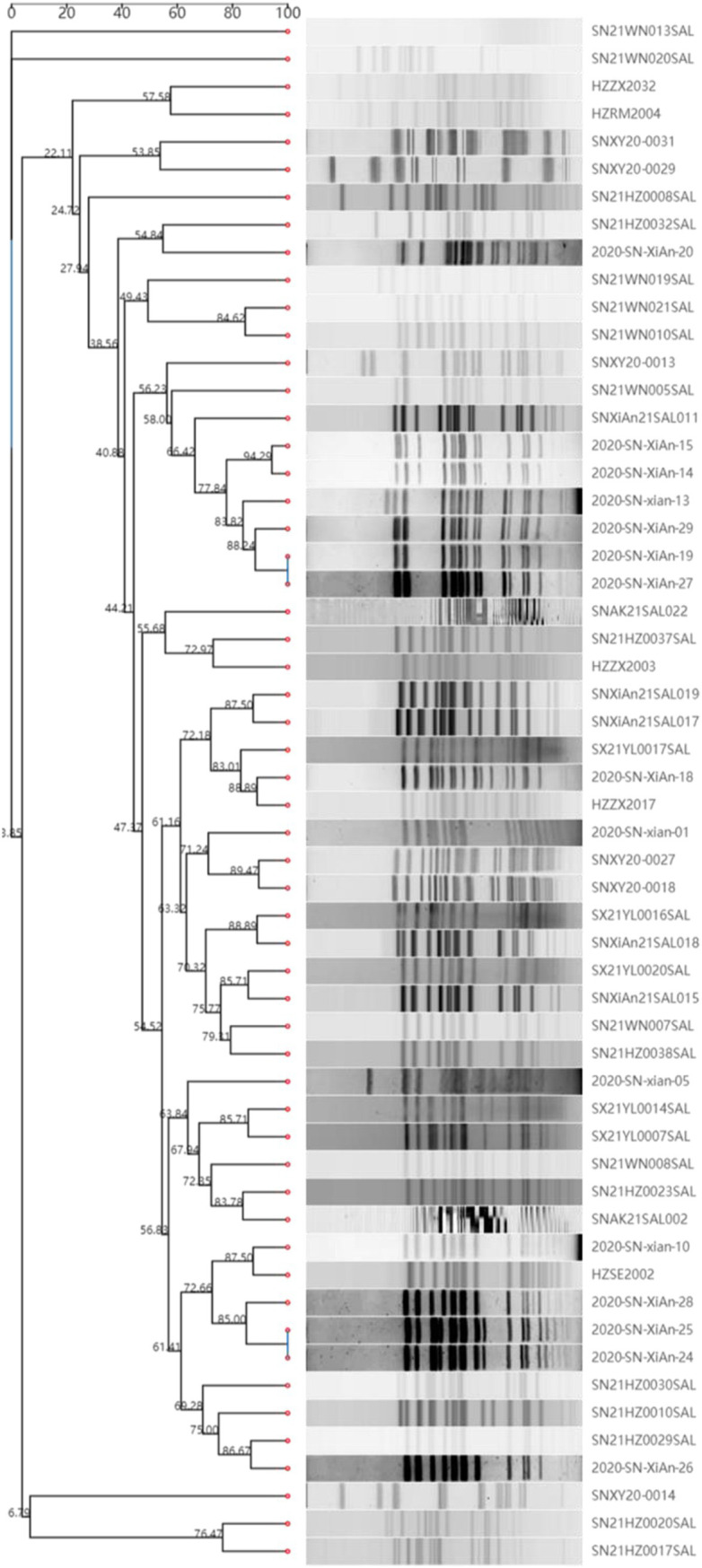
PFGE sequence types of 58 strains of monophasic *Salmonella* Typhimurium. The *Salmonella* serotype Braenderup strain (H9812) served as a molecular weight marker. The dendrogram was automatically generated online using the CCPIN platform.

To determine the genotype, PFGE and MLST were performed. The results revealed that the 58 strains of monophasic *Salmonella* Typhimurium exhibited significant diversity in PFGE subtypes (Figure 1). These findings suggest the possibility of multiple independent emergence events. However, MLST typing results showed that both the food isolates and the patient isolates belonged to the ST34 type.

### 3.3 Phenotypic antimicrobial resistance

To assess the most common patterns of antibiotic resistance, the phenotypic antimicrobial resistance of 53 successfully resuscitated monophasic *Salmonella* Typhimurium strains was evaluated against 17 antimicrobial agents belonging to 8 different classes. Antimicrobial resistance was classified as resistant, susceptible, or intermediate according to the guidelines established by the Clinical Laboratory Standard Institute. As presented in Table 2, the highest resistance rates were found in tetracycline and ampicillin (94.34%; 50/53), followed by streptomycin (88.68%; 47/53) and ampicillin/sulbactam at 64.15% (34/53). Additionally, the resistance rate to chloramphenicol and trimethoprim-sulfamethoxazole was both 47.17% (25/53). Additionally, we found three strains exhibiting resistance to colistin; however, none of these strains carried the *mcr* resistance gene.

**Table 2:** Antimicrobial susceptibility interpretation of the 53 isolated monophasic *Salmonella* Typhimurium strains.

Antibiotic agent	Breakpoint interpretive criteria (μg/ml)			Results in percentage (%)		
	Sensitive	Intermediate resistance	Resistant	Sensitive	Intermediate resistance	Resistant
Chloramphenicol	≤8	16	≥32	47.17(25/53)	5.66 (3/53)	47.17 (25/53)
Trimethoprim-sulfamethoxazole	≤2/38	–	≥4/76	52.83(28/53)	0 (0/53)	47.17 (25/53)
Colistin	-	≤2	≥4	0 (0/53)	94.34 (50/53)	5.66 (3/53)
Ertapenem	≤0.5	1	≥2	100 (53/53)	0 (0/53)	0 (0/53)
Meropenem	≤1	2	≥4	100 (53/53)	0 (0/53)	0 (0/53)
Cefotaxime	≤1	2	≥4	66.04 (35/53)	0 (0/53)	33.96 (18/53)
Ceftazidime	≤4	8	≥16	79.25 (42/53)	13.21 (7/53)	7.55 (4/53)
Ceftazidime / avibactam	≤8/4	–	≥16/4	98.11 (52/53)	1.89 (1/53)	0 (0/53)
Tetracycline	≤4	8	≥16	5.66 (3/53)	0 (0/53)	94.34 (50/53)
Tigecycline	≤0.5	–	>0.5	100 (53/53)	0 (0/53)	0 (0/53)
Ciprofloxacin	≤0.06	0.12–0.5	≥1	28.30 (15/53)	54.72 (29/53)	16.98 (9/53)
Nalidixic acid	≤16	–	≥32	66.04 (35/53)	0 (0/53)	33.96 (18/53)
Azithromycin	≤16	–	≥32	92.45 (49/53)	0 (0/53)	7.55 (4/53)
Amikacin	≤16	–	≥32	98.11 (52/53)	0 (0/53)	1.89 (1/53)
Streptomycin	≤8	16	≥32	5.66 (3/53)	5.66 (3/53)	88.68 (47/53)
Ampicillin	≤8	16	≥32	5.66(3/53)	0 (0/53)	94.34 (50/53)
Ampicillin / sulbactam	≤8/4	16/8	≥32/16	11.32 (6/53)	24.53 (13/53)	64.15 (34/53)

**Figure 2:**
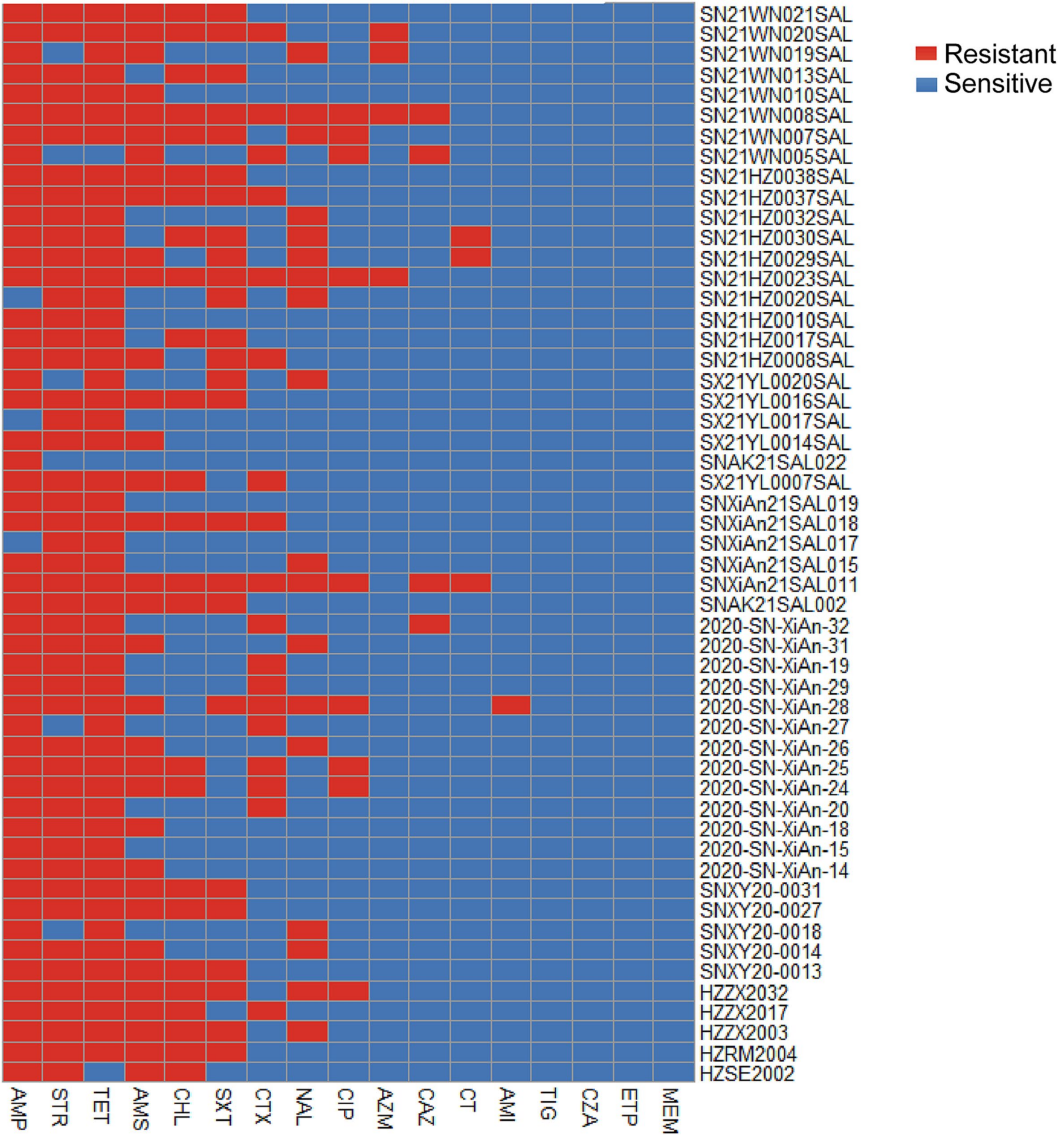
Heatmap of the phenotypic antimicrobial resistance of 53 isolated *Salmonella* strains. Red represents resistance, while blue indicates sensitivity. The heatmap was constructed based on the resistance similarity of each strain to 17 antimicrobial agents. CHL, Chloramphenicol; SXT, Trimethoprim-sulfamethoxazole; CT, Colistin; ETP, Ertapenem; MEM, Meropenem; CTX, Cefotaxime; CAZ, Ceftazidime; CZA, Ceftazidime / avibactam; TET, Tetracycline; TIG, Tigecycline; CIP, Ciprofloxacin; NAL, Nalidixic acid; AZM, Azithromycin; AMI, Amikacin; STR, Streptomycin; AMP, Ampicillin; AMS, Ampicillin / sulbactam.

When considering intermediate strains as susceptible, all strains were classified as resistant, as illustrated in Figure 2. Among the isolated strains, 26.42% (14/53) were resistant to five antimicrobial agents, while 24.53% (13/53) showed resistance to three agents, and 20.75% (11/53) were resistant to four agents.

### 3.4 Prediction of the antimicrobial resistance genes

**Figure 3:**
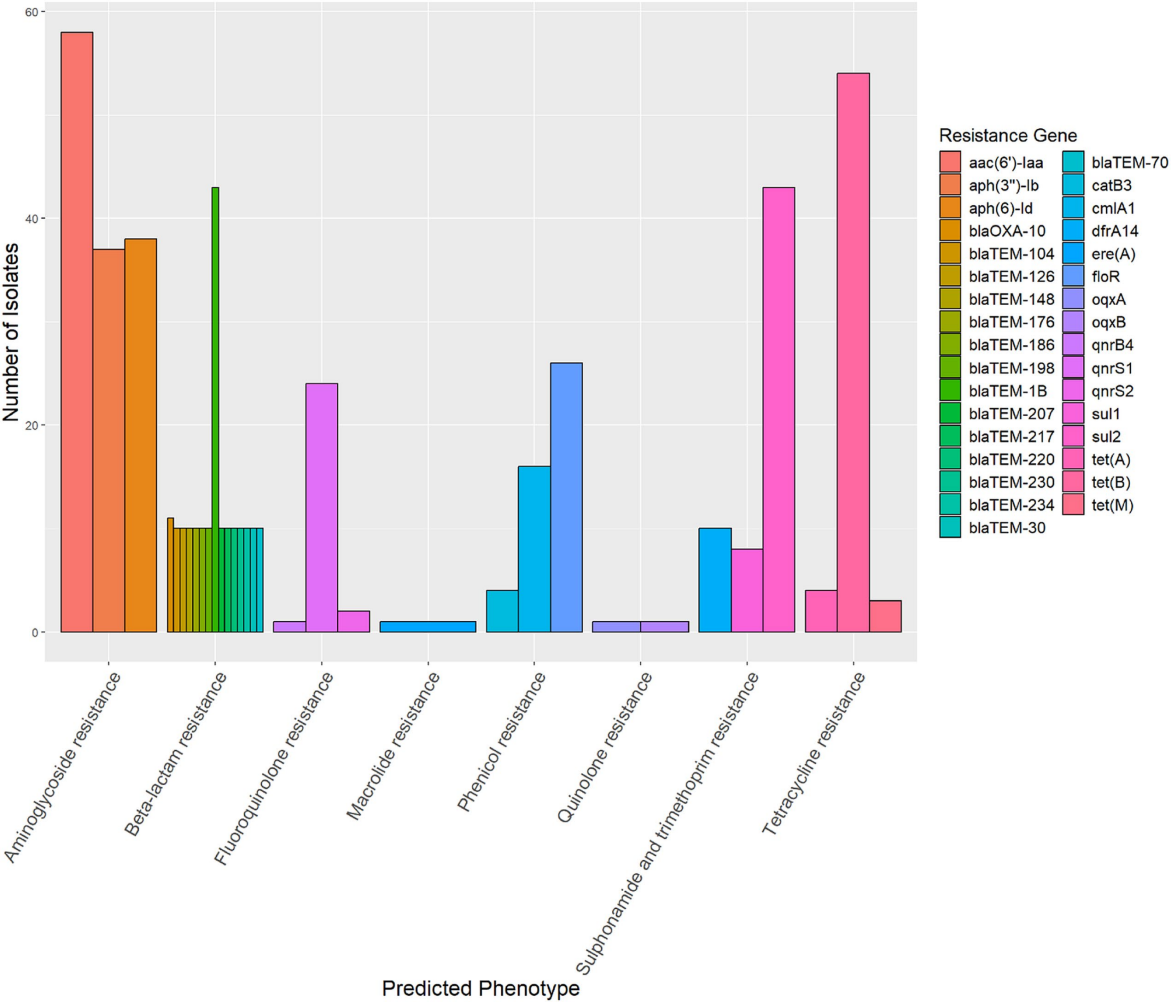
The prediction of antimicrobial resistance genes of 58 strains of monophasic *Salmonella Typhimurium*. There were 64 distinct genes associated with resistance to seven antimicrobial classes. The diagram illustrates the top three genes for each antimicrobial class.

The genomes of 58 strains were uploaded to the CCPIN platform for the prediction of antimicrobial resistance genes. The results indicated the presence of 64 distinct genes associated with resistance to 7 antimicrobial classes. Notably, *aac(6′)-Iaa* was found to be the most prevalent gene conferring resistance to aminoglycosides, detected in 100.00% (58/58) of the strains. This was followed by *tet(*B*)*, which conferred resistance to tetracycline in 93.10% (54/58) of the strains, *bla*_TEM-1B_, responsible for beta-lactam resistance, found in 74.14% (43/58), and *sul2*, which was resistant to trimethoprim-sulfamethoxazole (74.14%; 43/58). Additionally, the *floR* gene was present in 44.83% (26/58) of the strains, while *qnrS1* was detected in 41.38% (24/58) (Figure 3). We did not find mutations in the *gyrA*, *gyrB*, *parC*, and *parE* genes among the ciprofloxacin-resistant strains (data not shown).

### 3.5 Prediction of the virulence genes

To determine the virulence genes of *Salmonella*, the genomes of the 58 isolates were assessed with the virulence factors database. Among these, the *sodC1* gene was positive in 96.55% (56/58) strains, while the *sopE* gene was positive in 10.34% (6/58) strains (Table 3). No strains were found to carry the genes *sspH1*, *spvC*, *pefA*, or *rck*.

**Table 3:** The prediction of the virulence genes of 58 strains of monophasic *Salmonella* Typhimurium.

	*sspH1*	*sodC1*	*sopE*	*spvC*	*pefA*	*rck*
Absent	58	2	52	58	58	58
Present	0	56	6	0	0	0

### 3.6 Plasmid replicon predictions

The most prevalent plasmid replicon identified was IncQ1, detected in 84.48% (49/58) of the isolates, followed by IncHI2 and IncHI2A, each found in 32.76% (19/58) of the isolates. The strains carried between one and four types of plasmid replicons, and 8 of 58 isolates (13.79%) were found to harbor no plasmid replicons.

### 3.7 The phylogenetic relationship of isolates

**Figure 4:**
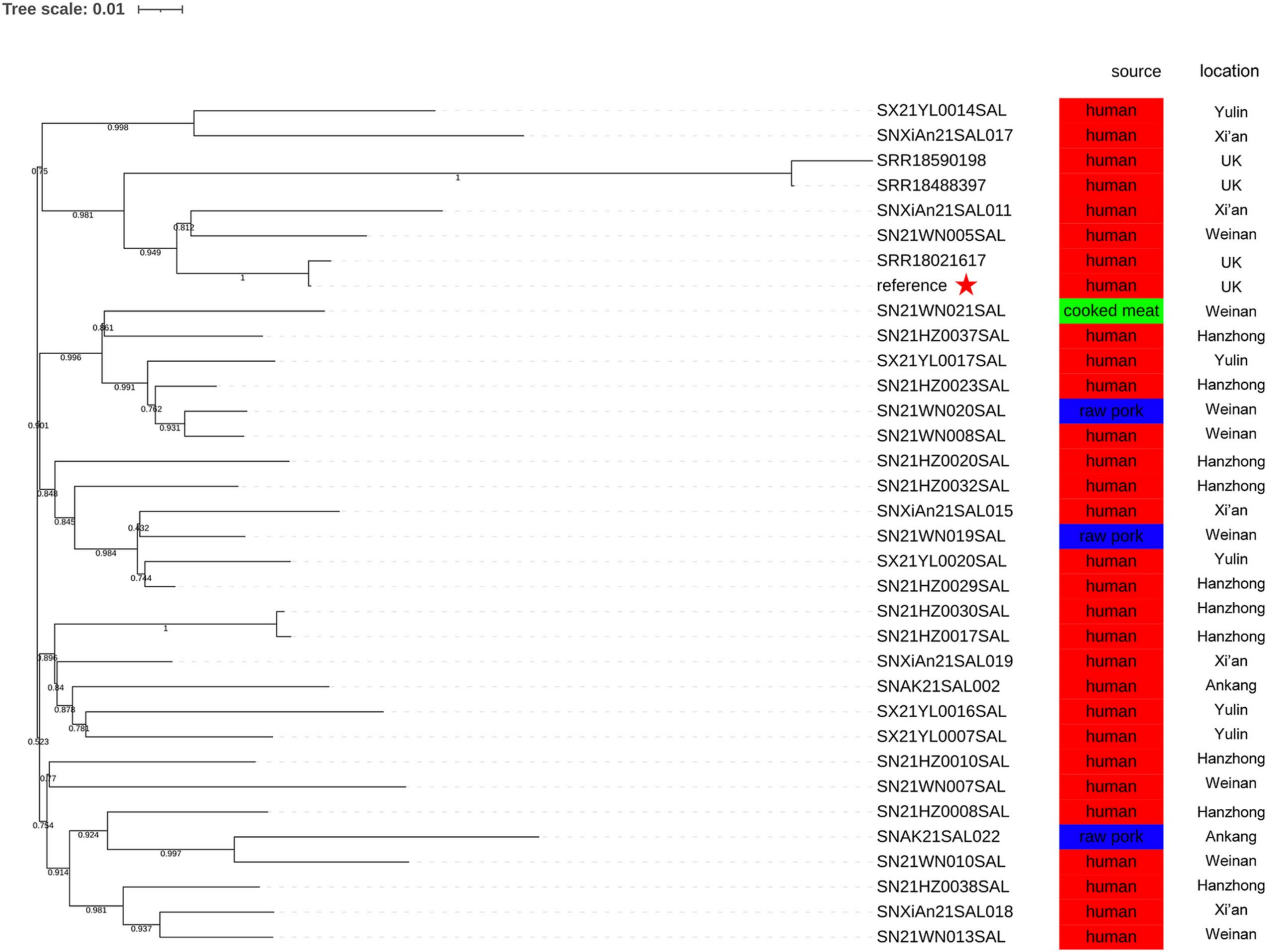
Phylogenetic relationship between 4 representative strains (SRR17830210, SRR18021617, SRR18488397, and SRR18590198) and 30 monophasic *Salmonella* Typhimurium strains isolated in 2021. The reference genome was SRR17830210. The geographical locations and sources are presented.

In this study, SRR17830210 served as the reference genome. The SNP analysis of 30 monophasic *Salmonella* Typhimurium strains isolated in 2021 and 4 representative strains revealed that there were 3 distinct clusters (Figure 4). The SN21WN005SAL, SNXiAn21SAL011, SNXiAn21SAL017, SX21YL0014SAL, and 4 representative strains were clustered together in the same branch with a bootstrap value of 0.750 (Figure 4). Meanwhile, SN21WN005SAL and SNXiAn21SAL011 were clustered together with the reference strain (SRR17830210) with a bootstrap value of 0.949 (not within five SNPs).

**Figure 5:**
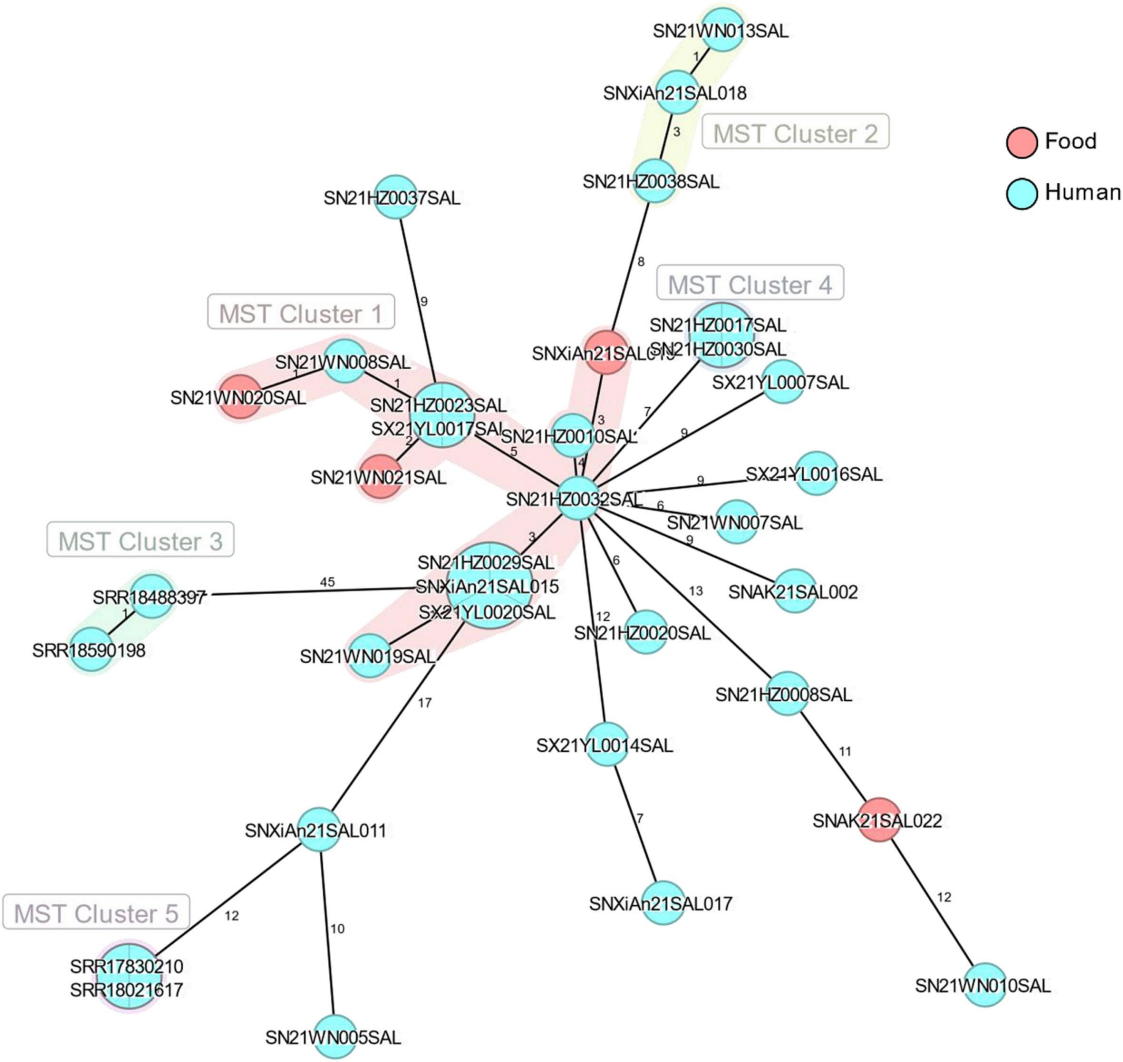
The minimum spanning tree of 4 representative strains (SRR17830210, SRR18021617, SRR18488397, and SRR18590198) and 30 monophasic *Salmonella* Typhimurium strains isolated in 2021. The numbers between strains represented allelic differences by using core genome MLST analysis. Light red circles represent strains from food sources, and cyan circles represent strains from human sources.

Using core genome MLST analysis, the difference between SNXiAn21SAL011, SRR17830210, and SRR18021617 was only 12 allelic differences (ADs), while SN21WN005SAL had a difference of 10 ADs compared to SNXiAn21SAL011 (Figure 5).

## 4 Discussion

There are multiple distinct clones of monophasic *Salmonella* Typhimurium, initially categorized into three geographical clones: the Spanish clone, the U.S. clone, and the major European clone. The Spanish clone, which is associated with pigs and pork products, is characterized by the deletion of the *fljA*, *fljB*, hin, and *iroB* genes, while the *STM2757* gene is conserved (Garaizar et al., 2002). The U.S. clone exhibits a unique genomic deletion pattern surrounding the *fljBA* operon but retains the *hin* and *iroB* genes (Soyer et al., 2009). The major European clone identified in 2005 is distinguished by the absence of the *fljA*, *fljB*, and *hin* genes, as well as the loss of the typical pSLT; however, it harbors conserved *iroB* and *STM2757* genes (Lucarelli et al., 2012). This clone primarily corresponds to ST34. Currently, the prevalence of the European clone has gradually surpassed that of the Spanish and U.S. clones (Elnekave et al., 2018). This study analyzed 58 strains of monophasic *Salmonella*. Among them, 4 strains originated from food, including 1 from cooked meat products and 3 from raw pork. The remaining 54 strains were isolated from patients. All 58 strains belonged to the ST34 type and lacked the *fljB* gene and virulence genes typically found on pSLT. However, only 12 strains possessed the STM2757 gene. Additionally, we utilized both PFGE and WGS to characterize the isolates. While WGS offers a comprehensive genomic understanding of relatedness, PFGE is a traditional molecular typing method that is widely used for outbreak strain tracing. The inclusion of PFGE allows us to compare our findings with a vast database of previous PFGE gel images, providing valuable context and validation for our results. Furthermore, the integration of these methodologies enhances our ability to detect genetic variations that could impact the epidemiological relationships between strains.

Previous research has indicated that *Salmonella* 4,[5],12:i:– isolates have a considerable diversity of subtypes, even among samples from a single country (Zamperini et al., 2007). It is suggested that this increased heterogeneity implies greater adaptability, which may contribute to the success of this serotype (Sun et al., 2020). In our study, the 58 strains of monophasic *Salmonella* Typhimurium demonstrated significant diversity in PFGE subtypes, suggesting that these isolates may differ considerably in their genetic backgrounds. While the lack of *fljB* expression is a notable trait among monophasic variants, the observed differences in PFGE patterns indicate that these variations could also arise from a range of genetic factors, including other gene deletions, mutations, or insertions unrelated to *fljB*. These insights highlight the necessity for further comprehensive genomic analyses to better understand the relationship between genetic deletions and the phenotypic characteristics of monophasic *Salmonella* Typhimurium.

The antibiotic resistance pattern of *Salmonella* serves as an important indicator of clonal evolution and lineage classification. The Spanish clone is resistant to up to 7 antimicrobial agents, including ampicillin, chloramphenicol, gentamicin, streptomycin/spectinomycin, sulfonamides, tetracyclines, and trimethoprim (ACGSSuTTp type). The ASSuT pattern, commonly observed in monophasic *Salmonella* Typhimurium in European strains from humans and pig products (Petrovska et al., 2016; Seixas et al., 2016), is also prevalent in swine and pork products in the United States, contributing significantly to the increase in multidrug-resistant *Salmonella* strains. This study revealed that tetracycline (94.34%; 50/53) and ampicillin (94.34%; 50/53) were the most resistant antimicrobial agents in Shaanxi Province, followed closely by streptomycin (88.68%; 47/53). This antimicrobial resistance pattern was similar to that of the ASSuT pattern. The antimicrobial resistance in the European clone may be attributed to the primary antibiotics used in pig breeding (Sun et al., 2020). Moreover, tetracycline and ampicillin are less effective antimicrobial agents against *Salmonella* isolates from the pig slaughtering process (Geng et al., 2021). These findings emphasize how antimicrobial use in livestock, particularly in pork production, may drive these patterns of resistance.

The European clones carry the same antimicrobial resistance genes, including *bla*_TEM_, *strA-strB*, *sul2*, and *tet(*B*)* (Hopkins et al., 2010; Seixas et al., 2016). In this study, our results showed that *aac(6′)-Iaa* was the most prevalent gene conferring resistance to aminoglycosides (100.00%; 58/58), followed by *tet(*B*)*, which expressed resistance to tetracycline (93.10%; 54/58), *bla*_TEM-1B_, which conferred resistance to beta-lactam antibiotics (74.14%; 43/58), and *sul2*, which was resistant to sulfonamides (74.14%; 43/58). Notably, *floR* (44.83%; 26/58) was identified as the gene with the highest prevalence of resistance to phenicols. Phenotypic resistance analysis revealed that the resistance levels for chloramphenicol and trimethoprim-sulfamethoxazole were comparable (47.17%; 25/53). It has been reported that the detection rates of *bla*_TEM-1B_, *sul2*, and *floR* are high in the pig slaughtering process (Geng et al., 2021). Additionally, the multidrug-resistant *Salmonella* DT104 also carries the *floR* gene (Switt et al., 2009). While our analysis has drawn comparisons to these three recognized clones, it is essential to recognize that the emergence of monophasic *Salmonella* Typhimurium is dynamic and multifaceted, with evidence of widespread genetic diversity. Numerous strains now exist that do not fit neatly into the original categories. Our study identified unique characteristics in the isolates from Shaanxi Province. This finding highlights the ongoing evolution of monophasic *Salmonella* Typhimurium and underscores the necessity for continuous surveillance and investigation of emerging strains.

Quinolones are essential antibiotics for the treatment of invasive *Salmonella* infections in humans (Cito et al., 2016). In He et al.’s (2020) study, 40.4% of monophasic *Salmonella* Typhimurium isolates were found to carry at least one plasmid-mediated quinolone resistance gene in China. The predominant genes identified were *oqxAB* and *aac(6′)-Ib-cr*, while the overall positive rate for the *qnr* gene was 7.8%. In this study, the prevalence of the *qnrS1* gene, which conferred reduced susceptibility to fluoroquinolones rather than full resistance, was found to be 41.38% (24/58). Additionally, one strain carrying the *blaCTX-M-14* gene was detected, which has been previously reported in a monophasic isolate collected from swine in Portugal (Clemente et al., 2013), and recovered from a human clinical sample (Fernandez et al., 2016). Notably, given that fluoroquinolones are generally contraindicated in children and pregnant women, ceftriaxone is often administered instead. This practice has implications for the treatment of infections, especially considering the presence of the *blaCTX-M* gene, which indicates potential resistance to cephalosporins.

Among the analyzed strains in our study, 96.55% (56/58) were positive for the virulence gene *sodC1*, while 10.34% (6/58) tested positive for *sopE*. The *sodC1* and *sopE* genes, located on prophages, exhibited comparable virulence capacities in comparison with *Salmonella* Typhimurium, causing human salmonellosis (Ngoi et al., 2018). The *sodC1* gene encodes a putative Cu/Zn superoxide dismutase (Ammendola et al., 2008), while SopE serves as one of the effector proteins that promote efficient bacterial entry into host cells (Rudolph et al., 1999). The virulence gene *sopE* was described in monophasic *Salmonella* isolates from Europe (Petrovska et al., 2016) and the United States (Elnekave et al., 2018).

The prediction results of plasmid replicons in 58 *Salmonella* isolates indicated that the predominant plasmid replicon was IncQ1 (84.48%; 49/58), followed by IncHI2 (32.76%; 19/58) and IncHI2A (32.76%; 19/58). It has been reported that IncQ1 is strongly associated with African multidrug-resistant *Salmonella* Enteritidis isolates (Cao et al., 2023). Wu et al. (2005) reported that IncHI2A_1 and IncHI2_1 were predominant in *Salmonella* Typhimurium ST34 isolates throughout the pig slaughtering process in China (Geng et al., 2021). It has been demonstrated that these plasmids were associated with resistance to different antimicrobial classes, including *β*-lactams, aminoglycosides, sulfonamides, tetracyclines, and polymyxins (Elbediwi et al., 2020a; Elbediwi et al., 2020b; Gu et al., 2020; McMillan et al., 2020). The virulence and antimicrobial resistance profiles of the isolates associated with these plasmids will be further studied.

Different pathogens show variations in SNP numbers and distributions due to differences in their genomic compositions. The SNP differences between outbreak strains may be related to the mutation rates and different outbreak patterns (such as geographic extent and period). The United Kingdom monophasic *Salmonella* Typhimurium outbreak reported a cluster of representative outbreak isolates within five ADs or five SNPs (European Centre for Disease Prevention and Control, European Food Safety Authority, 2022). In the present study, although the difference between SNXiAn21SAL011, SRR17830210, and SRR18021617 was 12 ADs, the SNP phylogenetic analysis showed that SN21WN005SAL, SNXiAn21SAL011, SRR17830210, and SRR18021617 were closely related, with a bootstrap value of 0.949. This result indicates the genetic similarities of the monophasic *Salmonella* Typhimurium strains. The metadata analysis revealed that all these isolates were from Shaanxi Province, with 4 strains sourced from food and the remaining from human cases. Notably, the genetic similarities of isolates from the same province could be attributed to shared environmental factors, local agricultural practices, and the transmission dynamics of *Salmonella*. Further research should explore these correlations to understand better the epidemiological aspects of the strains examined.

In this study, we investigated the genomic characterization and antibiotic resistance of monophasic *Salmonella* Typhimurium in Shaanxi Province, China. Although all strains belonged to sequence type ST34 and did not harbor virulence genes on the pSLT plasmid, 12 strains carried the STM2757 gene. The phenotypic assessment of antimicrobial resistance demonstrated that tetracycline, ampicillin, streptomycin, and trimethoprim-sulfamethoxazole were less effective antimicrobial agents. Moreover, the resistance levels of chloramphenicol and trimethoprim-sulfamethoxazole were found to be comparable. Gene prediction indicated that *aac(6′)-Iaa* was the most prevalent resistance gene, followed by *tet(*B*)*, *bla*_TEM-1B_, and *sul2*. Furthermore, *floR* exhibited the highest resistance among the phenicol-related genes. Though SNXiAn21SAL011, SRR17830210, and SRR18021617 were not within five ADs, the SNP phylogenetic relationships of SN21WN005SAL, SNXiAn21SAL011, SRR17830210, and SRR18021617 were closely related. This study not only contributes essential local molecular epidemiological insights but also underscores the urgent need for enhanced surveillance and strategic interventions to combat foodborne pathogens and optimize treatment options for salmonellosis in humans.

## Data Availability

The raw data supporting the conclusions of this article will be made available by the authors, without undue reservation.
